# Modification of physical properties of poly(L-lactic acid) by addition of methyl-β-cyclodextrin

**DOI:** 10.3762/bjoc.10.318

**Published:** 2014-12-16

**Authors:** Toshiyuki Suzuki, Ayaka Ei, Yoshihisa Takada, Hiroki Uehara, Takeshi Yamanobe, Keiko Takahashi

**Affiliations:** 1 Perkin-Elmer Japan Co. Ltd., 134 Godo-cho, Hodogaya-ku, Yokohama-shi 240-0004, Japan; Fax: +81-45-339-5861; 2 Gunma University, 1-5-1 Tenjin-cho, Kiryu-shi, Gunma 376-8515, Japan; Fax: +81-277-30-1331; 3 Tokyo Polytechnic University, 1583 Iiyama, Atsugi 243-0297, Japan; Fax +81-46-242-3000

**Keywords:** crystallinity, DSC, methyl-β-cyclodextrin, poly(L-lactic acid), Raman spectroscopy

## Abstract

Poly(L-lactic acid) (PLLA) is a biodegradable plastic and one of the most famous plastics made from biobased materials. However, its physical strength is insufficient compared to general-purpose plastics. In this study, the effect of methylcyclodextrin (MeCD) addition on the structure and physical properties, especially the drawing behavior, of PLLA was investigated. Through thermal analysis, it was found that MeCD addition lowers the crystallinity and enhances the mobility of PLLA. The sample containing approximately 17% MeCD was drawn to more than 1000% at 60 °C, although PLLA fractured at a strain of less than 100%. Differential scanning calorimetry (DSC)-Raman in situ measurements also revealed decreases in the glass transition temperature (*T*_g_), cold crystallization temperature (*T*_c_), and melting point (*T*_m_), and improvement in structural distribution with temperature. DSC-Raman measurements simultaneously supplied information about crystallinity and thermal properties. Thus, it was concluded that MeCD had high affinity for PLLA, and the addition of MeCD increased the amorphous component of PLLA and enhanced the drawability.

## Introduction

Poly(L-lactic acid) (PLLA) has attracted attention because it is a biodegradable polymer derived from carbon-neutral resources. However, its melting point (*T*_m_) of approximately 170 °C must be increased because of the low thermal resistance. The melting point of the stereo complex (Sc) of PLLA and poly(D-lactic acid) (PDLA) is higher than 220 °C [1–5], which is comparable to that of aromatic polyester. Many studies have been conducted for improving this thermal property using Sc [6–16].

PLLA is a brittle polymer, but the polymer should have a high toughness so that it can be used for mechanical purposes. Tensile drawing is one method of improving mechanical properties, since it effectively induces molecular orientation [17–27]. However, the brittleness of PLLA prevents drawing. To improve this, it is necessary to control the interchain interactions and lower the glass transition temperature (*T*_g_) of the amorphous phase.

Cyclodextrins (CDs) are cyclic molecules composed of six (α), seven (β), or eight (γ) glucose units. CDs have hydrophobic cavities that can contain guest molecules and form inclusion complexes (ICs). ICs with α-CD accelerate the nucleation and crystallization of poly(ε-caprolactone), poly(ethylene glycol), poly(butylene succinate), and poly(3-hydroxybutyrate) by acting as a nucleation agent [28–33].

Several attempts have been made to form ICs between CDs and PLLA, and their structures and physical properties have also been analyzed [34–36]. It has been reported that the loss factor peak, tanδ, shifted to a higher temperature because of IC formation [37]. In the solid ICs of CDs and PLLA, the CD–CD interactions are dominant over the PLLA–PLLA interactions. Therefore, by forming an IC, CD increases the *T*_g_ of PLLA, which is not desirable for the improvement of mechanical properties. Since this results from strong CD–CD interactions, a modified CD may improve the mechanical properties of PLLA. Methyl-β-cyclodextrin (MeCD) is soluble in chloroform, as is PLLA, and the affinity of MeCD for PLLA may be sufficient to improve the mechanical properties of PLLA.

In order to analyze the interchain interactions of polymers, it is necessary to acquire information about their thermal properties and structure. Differential scanning calorimetry (DSC) is a very useful method for analyzing the thermal properties such as *T*_g_ and cold crystallization temperature (*T*_c_). However, it does not provide details about the structural change. On the other hand, Raman spectroscopy reveals the vibrational states of functional groups, but not thermal properties because irradiation affects the sample temperature [38]. For exact and expeditious structural analysis, it is important to determine simultaneously the thermal properties and the local structure [39–40]. Recently, DSC and Raman spectroscopy (DSC-Raman), which simultaneously measure thermal behavior and Raman vibrational states, was developed. The purpose of this study is to investigate the effects of MeCD on the local structure and physical properties of PLLA by DSC-Raman spectroscopy.

## Results and Discussion

### Thermal properties

**Figure 1:**
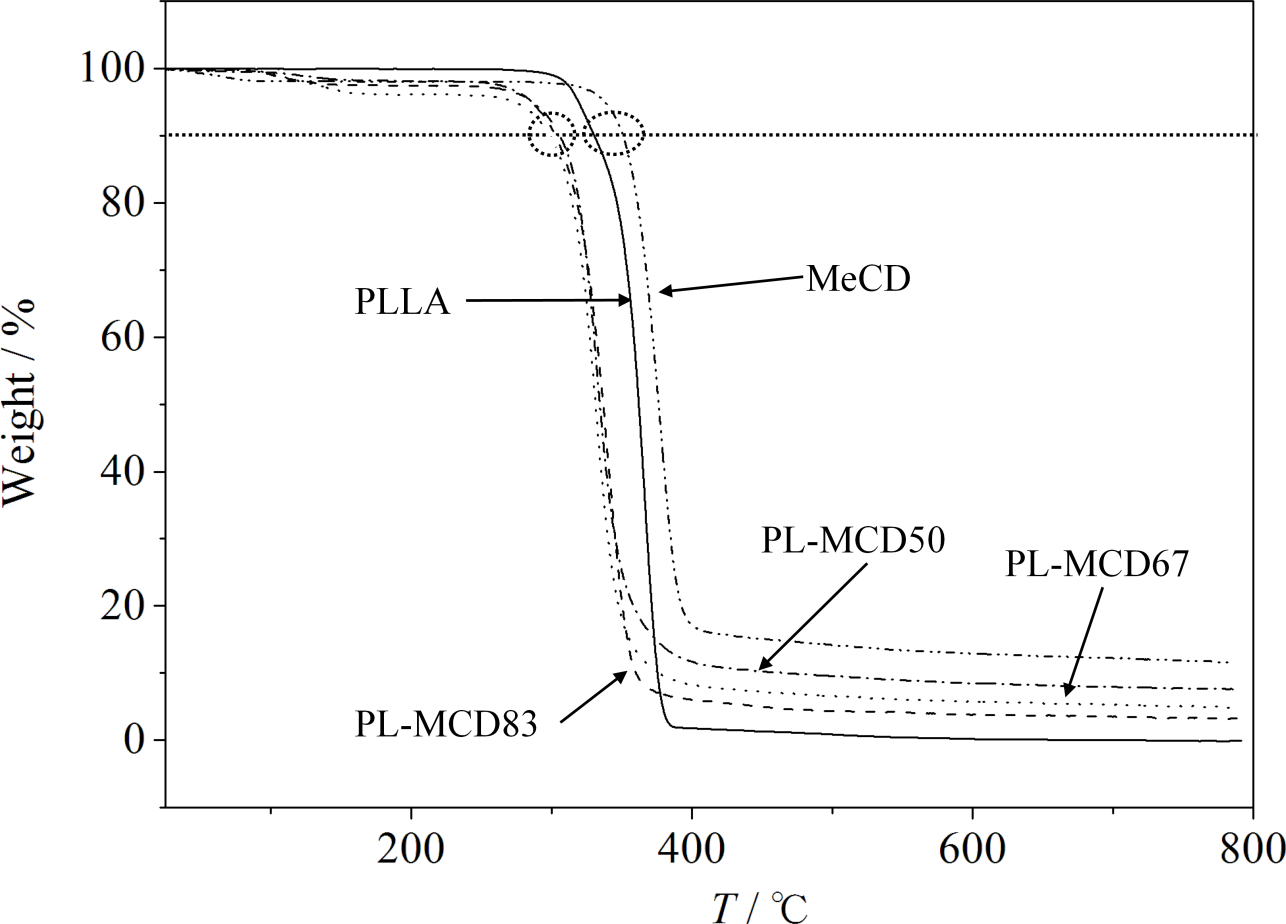
Thermo-gravitometry for PLLA, PL-MCD83, PL-MCD67, PL-MCD50 and MeCD.

Figure 1 shows the thermogravimetric analysis (TGA) results for MeCD, PLLA, PL-MCD83, PL-MCD67, and PL-MCD50. PL-MCD83, PL-MCD67, and PL-MCD50 refer to the mixtures of MeCD and PLLA with 83, 67, and 50 wt % of PLLA, respectively. Below 200 °C, a weight loss is observed, except for PLLA. For MeCD, this weight loss arises from dehydration [41–42]. For PL-MCD samples, the weight loss begins at higher temperatures than for MeCD. The weight loss is caused by desorption of chloroform from the MeCD cavity. This weight loss may not be observed if PLLA is included in the MeCD cavity, indicating that an IC with PLLA is not formed.

The 10% weight loss temperatures for PL-MCD50, 67, and 83 are lower than those of PLLA and MeCD. Since the decomposition is affected by crystallinity, the mixing of MeCD with PLLA lowers the thermal resistance because of the increase in the amorphous phase. In other words, MeCD causes PLLA to be disordered.

**Figure 2:**
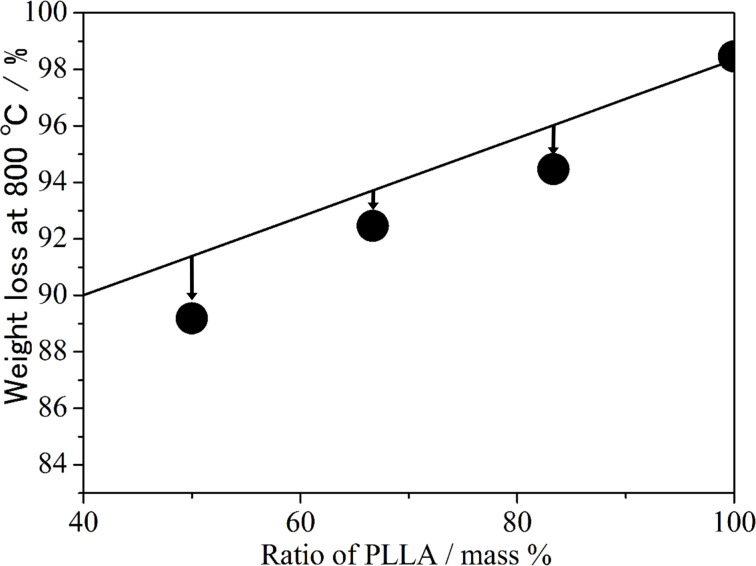
Observed weight loss and stoichiometric line.

Above 400 °C, most samples decompose, and at 800 °C, less than 12% of the residues of the decomposed samples remain. At 800 °C, PLLA and MeCD had 2 and 16 wt % of the residue, respectively. The decomposition weight loss of PLLA and MeCD mixture can be calculated from the linear relationship between the pure PLLA and MeCD if the interaction between MeCD and PLLA does not exist. Figure 2 shows the actual weight loss caused by decomposition at 800 °C and the theoretical weight loss line (the solid line). From this figure, it is clear that the actual weight loss is less than the theoretical weight loss. This means that the environment around the mixed MeCD and PLLA samples is different from that of pure PLLA and MeCD. Since the IC between PLLA and MeCD does not form according to the above results, the environmental changes around MeCD and PLLA may be caused by their high miscibility in each other. Therefore, MeCD acts as a plasticizer for PLLA. The plasticizing effects of triacetin and oligomeric poly(1,3-butylene glycol adipate) on PLLA have been reported [43–46]. Although these plasticizers are reported to be superior to MeCD, MeCD is a biobased material and is thus advantageous from an environmental perspective.

**Figure 3:**
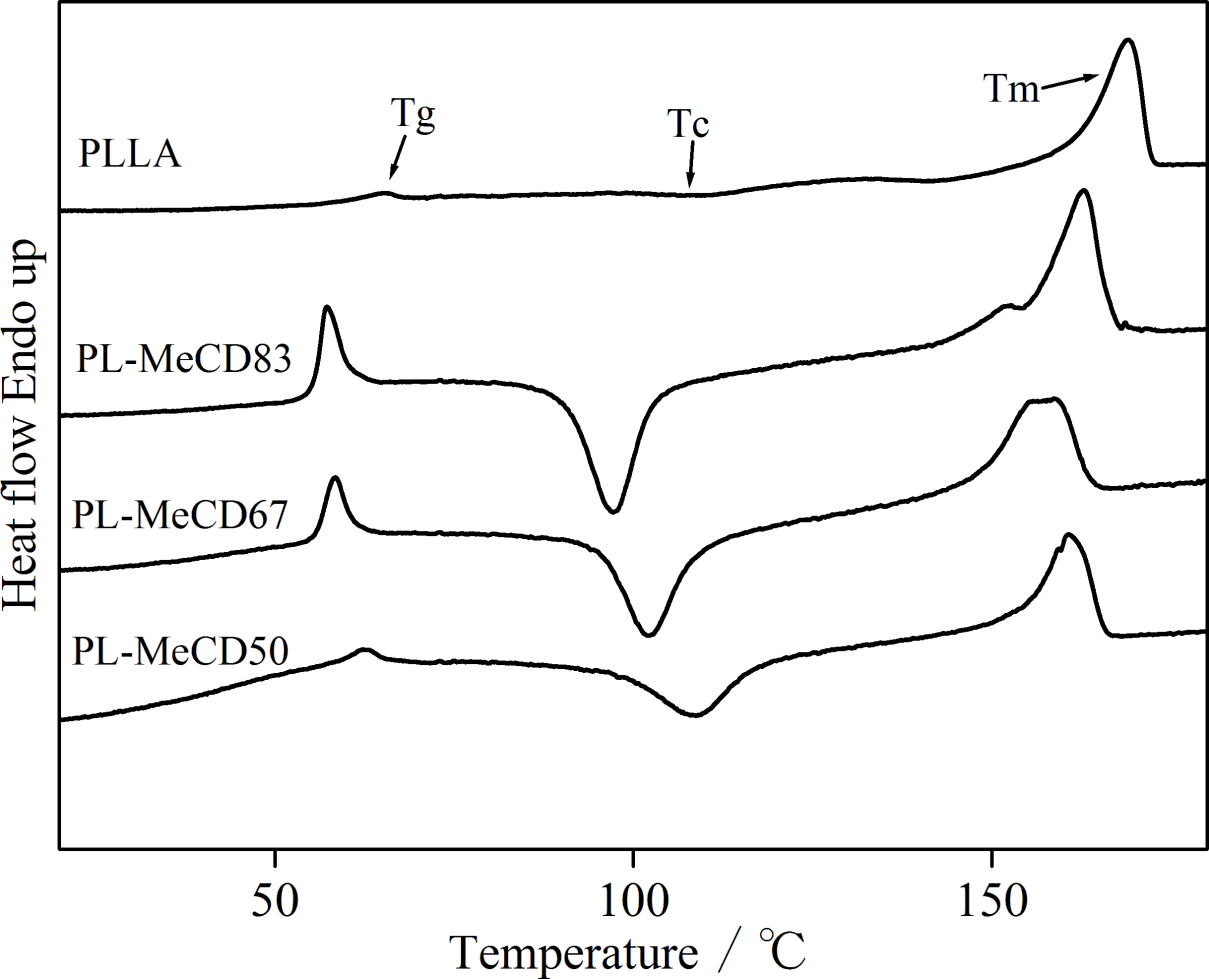
DSC curves for PLLA, PL-MCD83, PL-MCD67 and PL-MCD50.

These TGA results show that MeCD acts as a plasticizer for PLLA, and the fraction of the amorphous phase increases. Since the *T*_g_ of the samples may be affected by the MeCD addition, DSC measurements were also carried out. Figure 3 shows the DSC curves. For PLLA, the *T*_g_ and endothermal peak of melting are observed at approximately 70 and 180 °C, respectively. In addition, a slight cold crystallization is observed at approximately 120 °C [15,47–49]. Addition of MeCD alters the DSC curves. For PL-MCD83 and 67, *T*_g_ decreases to approximately 60 °C; similar trends were observed for *T*_c_ and *T*_m_. For PL-MCD50, *T*_g_ and *T*_c_ are higher than those of PL-MCD67 and 83, while their *T*_m_ is approximately equal. The enthalpies of fusion for PL-MCD50, 67, and 83 are approximately equal to the enthalpies of cold crystallization. Therefore, the melting of PL-MCD50, 67, and 83 are caused by the crystals formed through cold crystallization, and it can be concluded that PL-MCD50, 67, and 83 are amorphous. As the *T*_g_ and *T*_c_ decreased, the mobility of the amorphous phase increased through the addition of MeCD. It has been reported that the unmodified α-CD accelerates the crystallization of PCL, PEG, PBS and P3HB during cooling from melts. Cooling measurements by DSC were carried out for PLLA, PL-MCD50, 67 and 83 to confirm the similar effects. However, the crystallization was not observed for PLLA, PL-MCD50, 67 and 83 with a cooling rate of 10 °C·min^−1^. Therefore, MeCD does not have a potential to accelerate the crystallization of PLLA.

### Tensile drawing behavior

**Figure 4:**
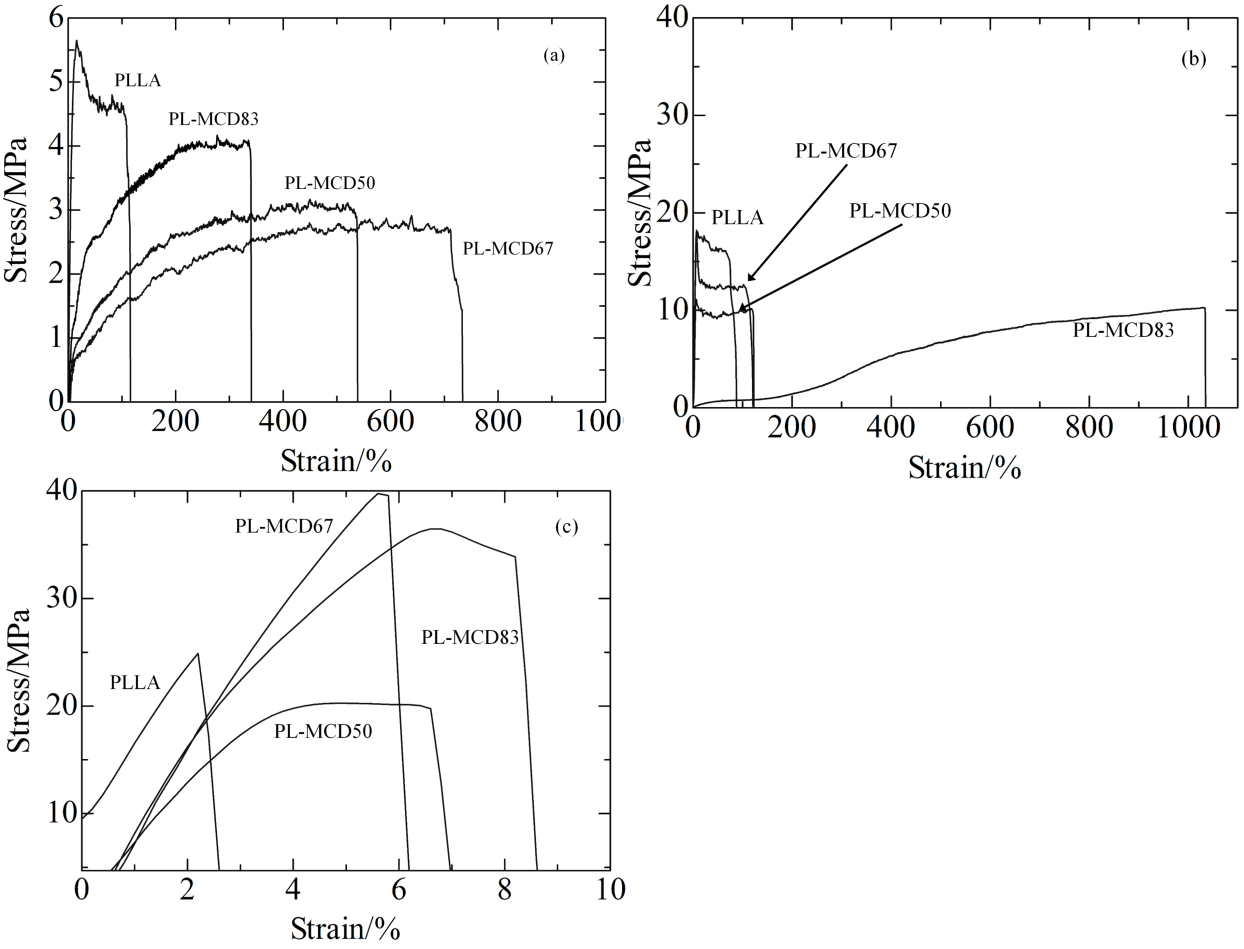
Stress-Strain curve for PLLA, PL-MCD50, 67 and 83 at a) 100 °C, b) 60˚C and c) 25 °C.

Figure 4 shows the stress-strain curves for the PLLA and PL-MCD samples at 25, 60, and 100 °C. At 25 °C, all samples fractured at less than 20% elongation in Figure 4c. The breaking stress at 25 °C is the highest for all the temperatures observed. This behavior is typical for brittle plastics, indicating that the *T*_g_ of the amorphous phase is higher than 25 °C.

At 60 °C, PLLA fractured at less than 100% strain, which is much larger than at 25 °C in Figure 4b. For PL-MCD50 and 67, similar behavior to PLLA was observed. These samples exhibited yield points, followed by extensive elongation at an almost constant strain, which is typical for a tough plastic where the semicrystalline polymer shows an amorphous phase above *T*_g_. In contrast, PL-MCD83 was drawn to a maximum strain of greater than 1000% and did not exhibit a yield point. The stress is low at short elongation and increases with elongation, which is typical for elastomers. Therefore, the mechanical properties of these samples are governed by rubber elasticity. In other words, MeCD prevents PLLA crystallization and lowers the *T*_g_ for PL-MCD83. In fact, the *T*_g_ of PL-MCD83 is lowest among the samples studied. This behavior is in accord with the TGA and DSC results.

At 100 °C, the stress-strain curves did not exhibit yield points except for PLLA in Figure 4a, since it contains a crystalline phase. The other samples behave as elastomers; the largest break strains are lower than those at 60 °C. This indicates that the mobility of PLLA molecules is high at 100 °C and that the tensile stress cannot propagate properly.

**Figure 5:**
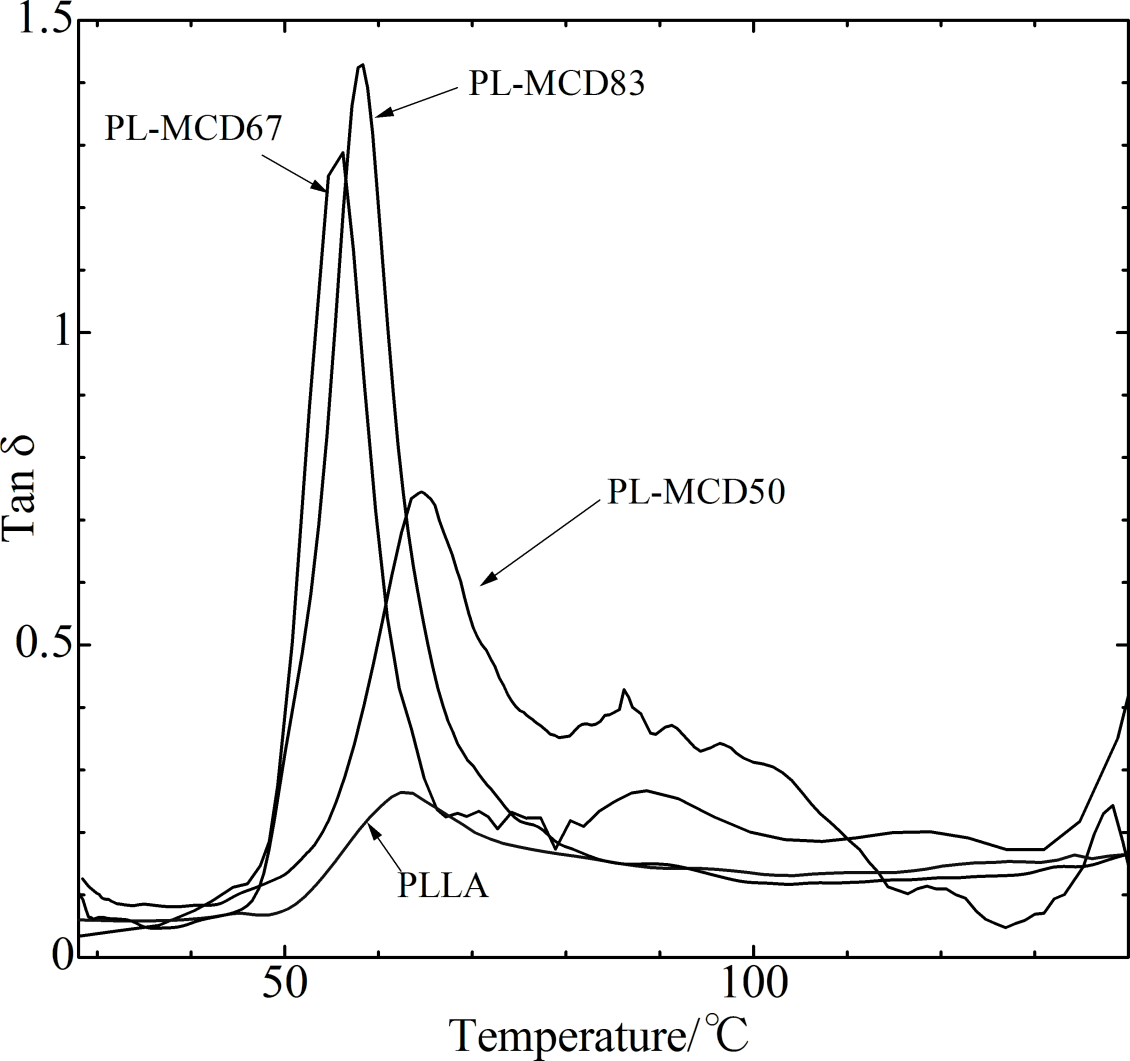
Temperature dependence of tanδ for PLLA, PL-MCD50, PL-MCD67 and PL-MCD83.

In addition, dynamic mechanical analysis (DMA) measurements were conducted. Figure 5 shows the temperature dependence tanδ. The peak assigned to the *T*_g_ for PLLA is approximately 70 °C [50]. For PL-MCD83 and 67, it is approximately 60 °C. Thus, it can be concluded that MeCD addition lowered the *T*_g_’s.

### DSC-Raman measurements

**Figure 6:**
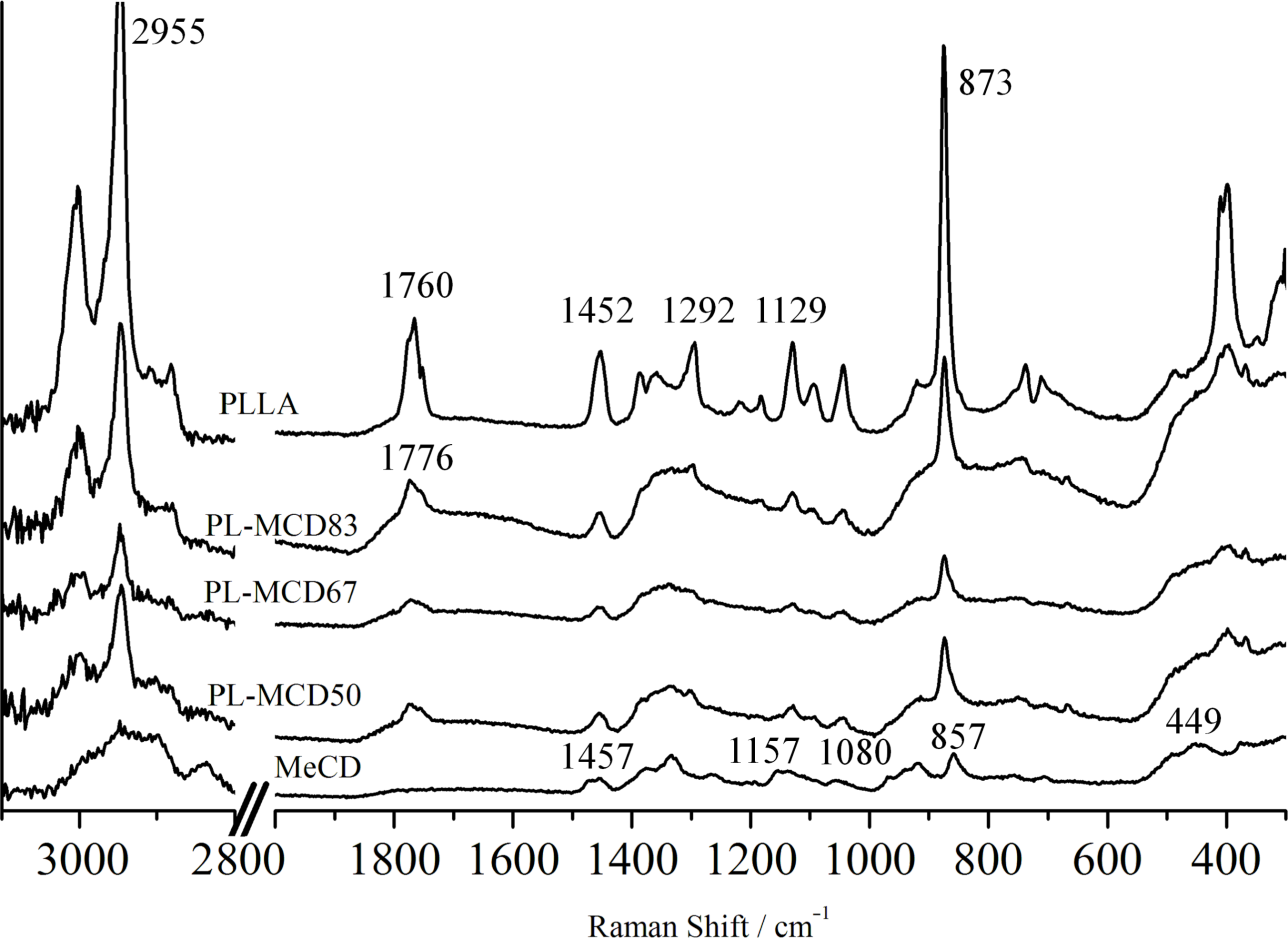
Raman spectra for PLLA, PL-MCD83, 67, 50 and MeCD at room temperature.

The Raman spectra of PLLA, PL-MCD50, 67, 83, and MeCD at 25 °C are displayed in Figure 6. The peak assignments of PLLA are summarized in Table 1. MeCD exhibited scattering peaks at 1457, 1157, 1080, 857, and 449 cm^−1^. High-intensity characteristic peaks for PLLA are at 2955 cm^−1^ (νCH), 1760 cm^−1^ (νC=O), 1452 cm^−1^ (δCH_3_), 1292 cm^−1^ (δCH), and 873 cm^−1^ (νC-Cα) [51–52]. Peaks at 857 and 449 cm^−1^ are characteristic for MeCD [53].

**Table 1:** Peak assignment of PLLA.

Peak assignment	Wave number [cm^−1^]

νCH_3_ as	2998
νCH,νCH_3 S_	2945, 2900
νC=O	1762
δCH_3_ as	1452
δCH_3_ s,δCH	1385, 1365, 1292
γCH	1215, 1180
γCH_3_	1129, 1091
νC_α_-C_β_	1042
νC_Ester_-C_α_,νC-C_α_	920, 872
γC=O	735, 707
τC-O_E,_ δO=CO_E_	510, 408, 394, 340
δCC=O, γCCC	305, 237

In the Raman spectra of PL-MCD50, 67, and 83, the PLLA peaks are clearly observed, while MeCD peaks are obscured. This is caused by peak overlap and weak MeCD peak intensity owing to the lower molar ratio of MeCD. Since the characteristic peak at 857 cm^−1^ for MeCD overlapped with the peak at 873 cm^−1^, it is difficult to analyze the structure of MeCD through this peak. Although the other characteristic peak of MeCD at 449 cm^−1^ is not typically employed, its intensity can be used to measure the state of MeCD, since PLLA does not exhibit a peak in this region.

The peak at 1760 cm^−1^ for PLLA shifts to a higher wavenumber by approximately 6 cm^−1^ because of MeCD addition. This peak shift is likely caused by the interaction between PLLA and MeCD. Compared to the amorphous phase, the wavenumber of the Raman peak for the crystalline phase is lower by 6 cm^−1^ [54]. Therefore, the addition of MeCD induces the amorphous phase of PLLA. The shift of the peak at 1760 cm^−1^ indicates the change in crystallinity. For other peaks, a peak shift was insignificant. Although MeCD is mixed with PLLA, the IC was seemingly not formed. As in the tensile drawing test, PL-MCD83 exhibited the largest strain. Its structure was investigated through DSC-Raman measurements.

**Figure 7:**
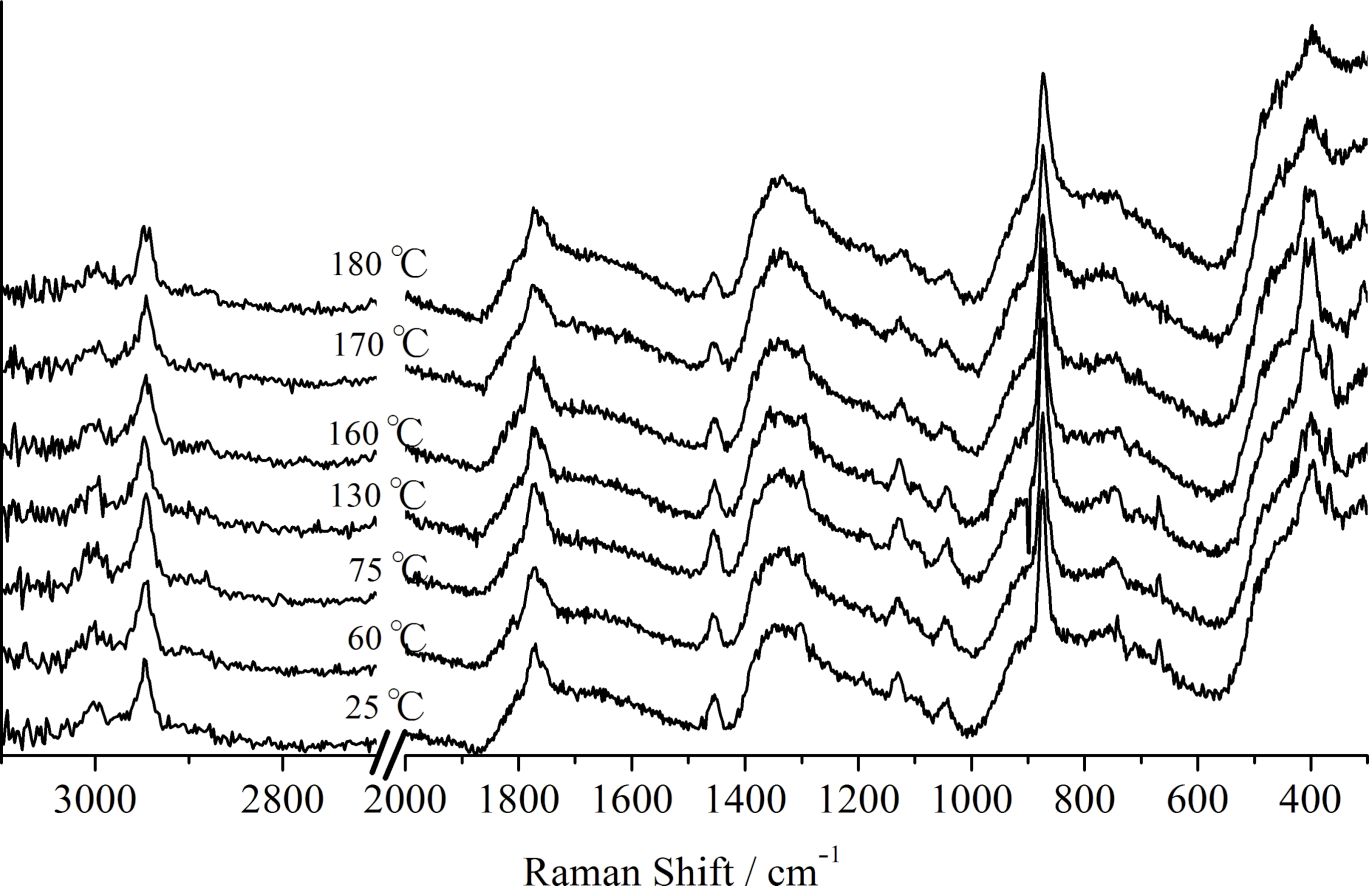
Temperature dependence of Raman spectrum of PL-MCD83.

Figure 7 shows the Raman spectrum of PL-MCD83 at a temperature close to its *T*_g_, *T*_c_, and *T*_m_. The peak intensities at 1760 and 873 cm^−1^, which are the characteristic peaks of PLLA, decreased as the temperature increased. In addition, both peaks broadened with temperature, which is remarkable above *T*_c_. Peak broadening and decrease in the peak intensity were also observed for other peaks.

**Figure 8:**
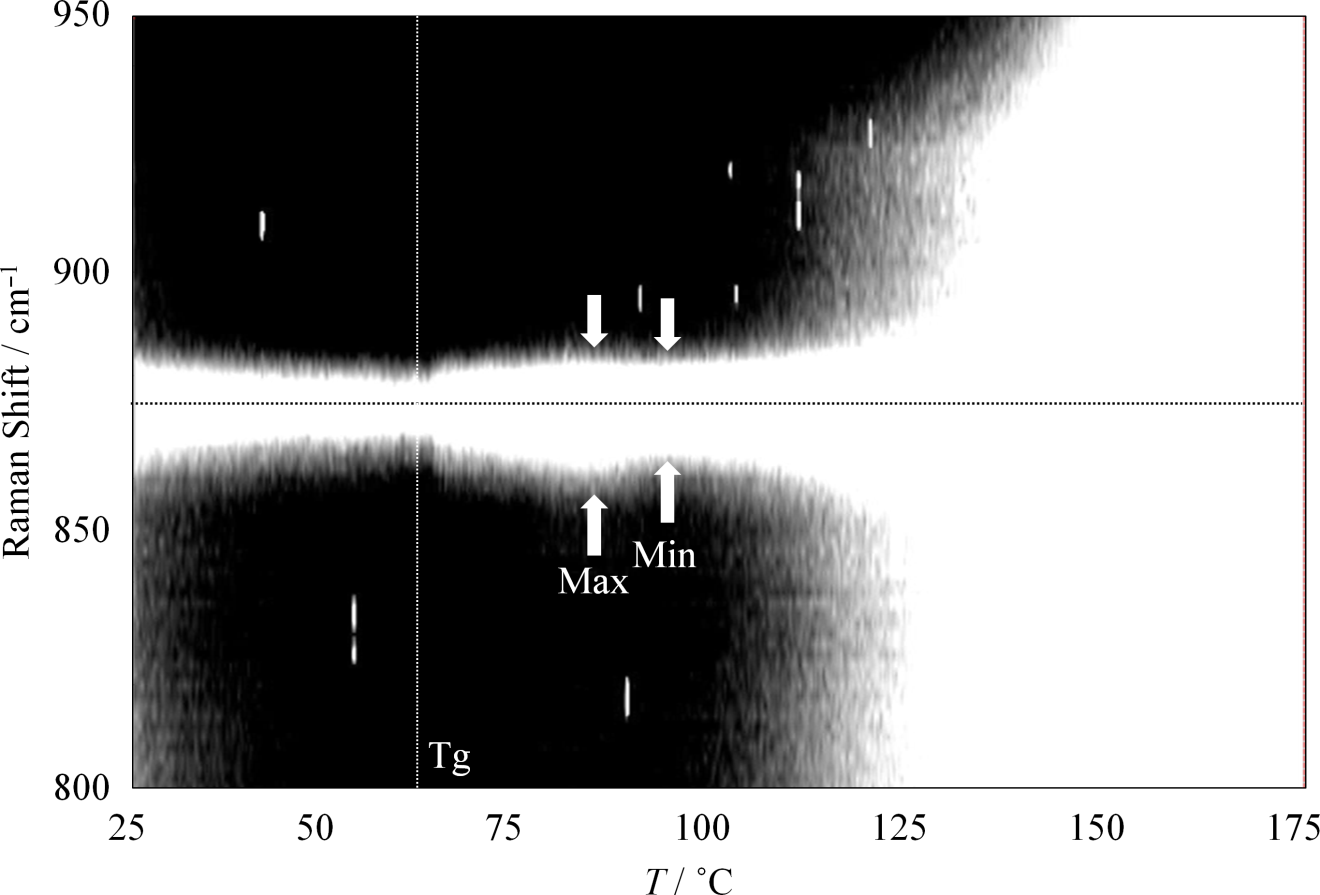
Stacked line profiles of Raman spectra of PL-MCD83 for the characteristic peak of PLLA at 873 cm^−1^.

The peak position, intensity, and width provide important structural information. Figure 8 shows the stacked line profiles of the Raman spectra of PL-MCD83 for the characteristic peak of PLLA at 873 cm^−1^. The horizontal dotted line represents the top of the peak at 873 cm^−1^. In this figure, the white region indicates high intensity. Therefore, the vertical width of the white region corresponds to the peak width. As the temperature increases from room temperature to 60 °C, the peak width either remains nearly constant or slightly decreases. Above the *T*_g_, the peak width increases and reaches a maximum at 80 °C. Between the *T*_g_ and 80 °C, the activation of segmental motion induces local structural distribution. In this temperature range, PLLA is in the amorphous state, and the molecular mobility is suitable to be drawn. Therefore, the largest strain was obtained for PL-MCD83 in this temperature range. Above 80 °C, the peak width begins to decrease, passes the minimum at approximately 100 °C, and then increases again. This sample undergoes cold crystallization at approximately 100 °C, causing peak narrowing at above 80 °C. The produced crystalline phase prevents drawing above *T*_c_, where the peak width monotonically increases. The peak broadening indicates that the local structure of the sample is disturbed by the thermal energy. Thus, in addition to the thermal properties, the local structural distribution can also be deduced from the DSC-Raman measurements. Furthermore, the tensile drawing behavior can be explained through molecular mobility and local structural distribution.

### Transition analysis by continuous intensity change of Raman spectrum

Since the peak area is proportional to the width and intensity, the peak intensity is also a good indicator for the local structure. Principle components analysis (PCA) is one of the most well-known techniques for analyzing intensity change in continuous data [55–56]. DSC-Raman results can be divided into elements of time, temperature, Raman scattering, and heat flow [57–58]. Since there was a one-to-one correspondence between the time and temperature in DSC-Raman, parameters extracted from temperature or time and intensities of scattering can be applied to transition analysis. This method has the advantage of being able to analyze the precise temperature dependence of each peak, and by extension, each functional group.

**Figure 9:**
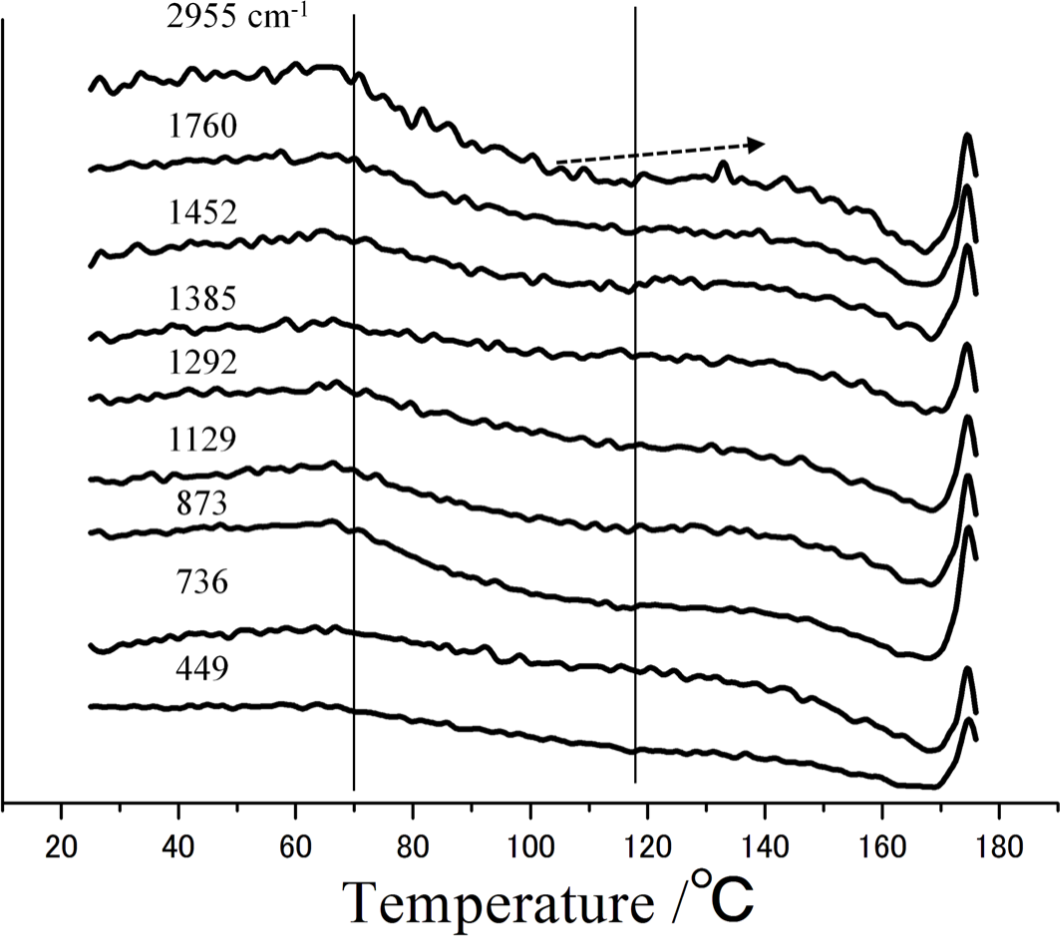
PCA results of PLLA at 2955 cm^−1^, 1760 cm^−1^, 1452 cm^−1^, 1385 cm^−1^, 1292 cm^−1^, 1129 cm^−1^, 873 cm^−1^, 736 cm^−1^ and 449 cm^−1^.

Figure 9 shows the PCA results for PLLA from simple integration of its characteristic peaks. The peak intensity at 2955 cm^−1^ is nearly constant from room temperature to 70 °C. Above 70 °C, the intensity decreases suddenly. This decrease corresponds with the *T*_g_. In the DSC curve, the *T*_g_ of PLLA appeared as a slight baseline change because this sample was not amorphous. In Figure 9, the intensity change at *T*_g_ is clear for PLLA. Therefore, PCA analysis of DSC-Raman measurements can detect thermal changes with high sensitivity.

The peak intensity decreases between 70 and 120 °C. Above 120 °C, the intensity increases slightly, which corresponds to cold crystallization. Thus, the behavior of the peak intensity is similar to the DSC heat flow. The intensity change of other peaks is similar to that of the peak at 2955 cm^−1^.

**Figure 10:**
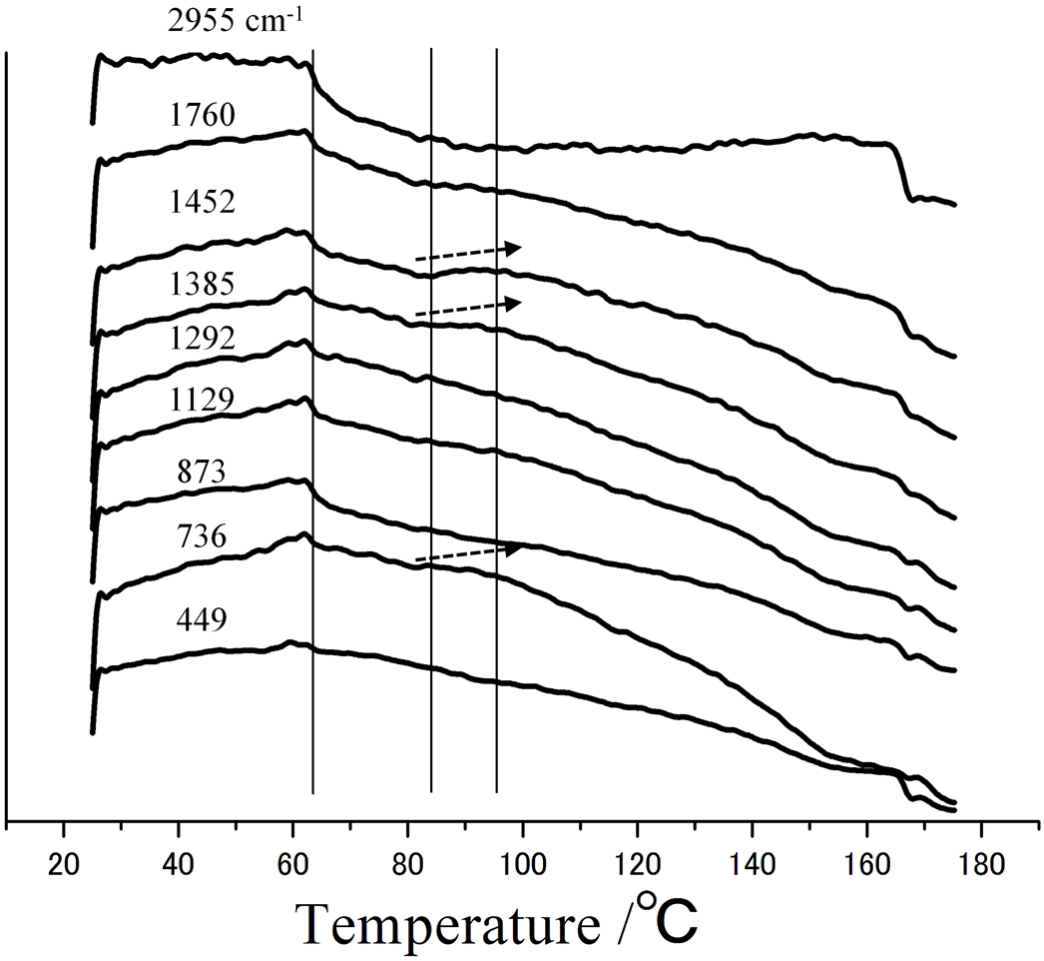
PCA results of PL-MCD83 at 2955 cm^−1^, 1768 cm^−1^, 1452 cm^−1^, 1385 cm^−1^, 1294 cm^−1^, 1128 cm^−1^, 873 cm^−1^, 736 cm^−1^ and 449 cm^−1^.

Figure 10 shows the temperature dependence of the peak intensities for PL-MCD83. The peak intensities decreased clearly at approximately 60 °C, which corresponds to the *T*_g_. The *T*_g_ of this sample is lower than that of PLLA, which is in accord with the deterioration of the crystalline phase from MeCD addition.

The peaks at 1385 and 1452 cm^−1^ increase at approximately 100 °C. The DSC data clearly indicate that cold crystallization occurred at this temperature. Thus, the increase in the peak intensities at 100 °C corresponds to cold crystallization. This temperature was lower than that for PLLA. These results, along with DSC-Raman measurements, indicate that MeCD addition lowers the *T*_g_ and *T*_c_.

The change in peak intensity at 449 cm^−1^ was similar to those of PLLA peaks. Since MeCD is the main contributor to this peak, this behavior might be affected by the structure of MeCD. At *T*_g_, this peak also decreased, which indicates that the mobility of MeCD increased with the segmental motion of PLLA. Above *T*_g_, the peak intensity at 449 cm^−1^ monotonically decreases. The temperature dependence of this peak is similar to that of PLLA over the temperature range observed. Therefore, MeCD has good affinity for PLLA over a wide range of temperatures. Thus, the analysis of DSC-Raman spectra reveals not only the thermal behavior but also the local structural information of each component with high sensitivity.

## Conclusion

The effect of MeCD addition to PLLA on its physical properties was examined. As revealed through TGA and DSC, the addition of MeCD lowers the *T*_g_, *T*_c_, and *T*_m_. These results indicate that MeCD prevents PLLA crystallization and increases the amorphous content while enhancing molecular mobility in the amorphous phase. This fact was reflected in the tensile drawing results, in which PL-MCD83 exhibited the largest strain at 60 °C. Since the affinity of MeCD for PLLA is high, MeCD may potentially be used as a plasticizer for PLLA. DSC-Raman measurements showed results similar to those of TGA, DSC, tensile drawing, and DMA. In addition, the detailed behaviors of PLLA and MeCD during heating were observed. DSC-Raman offers detailed information regarding the thermal behavior and local structure simultaneously, and is useful for precise temperature-dependent local structure analysis.

## Experimental

### Materials

MeCD was purchased from Wako Pure Chemical Ind. (Japan) and used as received. In this MeCD, two methoxy groups per glucopyranose unit were introduced in β-CD. PLLA (LACEA, Mitsui Chemicals Inc.; *M*_n_ = 1.3 × 10^5^, *M*_w_ = 2.6 × 10^5^) was used after removing the oligomer and polymerization catalyst by dissolution in chloroform and precipitation in methanol. The precipitated PLLA was dried at 100 °C for 24 h in vacuo prior to film preparation. Chloroform and methanol were purchased from Wako Pure Chemical Ind. (Japan) and used as received.

### Sample film preparation

Sample solutions of 1 wt % PLLA and MeCD were prepared separately by dissolving the requisite amounts of PLLA and MeCD in chloroform at room temperature. The solutions were mixed and vigorously stirred for 24 h. The PLLA/MeCD chloroform solutions were cast onto a Petri dish and covered with an aluminum foil with holes to allow solvent evaporation at room temperature. The neat PLLA film was prepared with same way. The obtained films were dried in vacuo. Casted films with MeCD/PLLA ratios of 1:1 (50 wt % PLLA), 1:2 (67 wt % PLLA), and 1:5 (83 wt % PLLA) were prepared. The samples were abbreviated as PL-MCD and the wt % values of PLLA were written after the abbreviated name (e.g., PL-MCD50 for MeCD/PLLA = 1:1). The obtained films were used for the experiments; for MeCD, the as received powder was used.

### Thermogravimetry/differential thermal analysis (TG-DTA)

TG-DTA (Perkin-Elmer STA6000) was carried out at 10 °C min^−1^ under a nitrogen flow of 60 mL min^−1^ with 5 mg samples placed in an open Al_2_O_3_ pan. For the stability test and accuracy of complex mass ratio, the measurements were carried out from 25 to 800 °C. MeCD was used as received.

### Tensile drawing test

Tensile drawing of samples was carried out at 25, 60, and 100 °C using a Tensilon RTC-1325A tensile tester (A&D, Japan). The initial sample length was 10 mm. Tensile drawing measurements were carried out at a cross-head speed of 20 mm min^−1^.

### Dynamic mechanical analysis (DMA)

DMA was conducted from 25 to 140 °C at 10 °C min^−1^ at a frequency of 1 Hz under a nitrogen atmosphere using DMA861e (Mettler, Toledo). Samples were cut into 25.0 mm × 3.0 mm films.

### Constant rate DSC measurements

For thermal behavior analysis, the constant rate DSC measurements were carried out using a power compensation DSC (Perkin-Elmer DSC 8500). Samples (2 mg) were packed in an Al pan and the measurements were carried out at 10 °C min^−1^ under a nitrogen flow of 20 mL min^−1^. MeCD was used as received.

### DSC-Raman measurements

A power compensation DSC (Perkin-Elmer DSC 8500) connected to a Raman spectrometer (Perkin-Elmer Raman Station 400) through a DSC-Raman interface probe was used. The laser irradiation of the samples and scattering collection from the samples were conducted through glass fibers to minimize the laser irradiation area and to avoid temperature changes. Laser irradiation was configured to 100 mW for 4 s with a heat flow amplitude of less than 8 mW or 0.03 °C [59]. The collection of Raman spectral data ranged from 3200 to 200 cm^−1^. DSC-Raman measurements were carried out from 25 to 190 °C at a heating rate of 2 °C min^−1^. The samples (4 mg) were placed in an Al pan, and a SiO_2_ disk was placed on the sample to minimize the effect of sample thickness on Raman intensity. MeCD was used as received.

## References

[1] Ikada Y, Jamshidi K, Tsuji H, Hyon S-H (1987). Macromolecules.

[2] Tsuji H, Hyon S-H, Ikada Y (1991). Macromolecules.

[3] Tsuji H, Hyon S-H, Ikada Y (1991). Macromolecules.

[4] Tsuji H, Ikada Y (1993). Macromolecules.

[5] Tsuji H, Ikada Y (1999). Polymer.

[6] Tsuji H, Ikada Y, Hyon S-H, Kimura Y, Kitao T (1994). J Appl Polym Sci.

[7] Takasaki M, Ito H, Kikutani T (2003). J Macromol Sci, Part B: Phys.

[8] Furuhashi Y, Kimura Y, Yoshie N, Yamane H (2006). Polymer.

[9] Tsuji H, Nakano M, Hashimoto M, Takashima K, Katsura S, Mizuno A (2006). Biomacromolecules.

[10] Ishii D, Ying T H, Mahara A, Murakami A, Yamaoka T, Lee W-k, Iwata T (2009). Biomacromolecules.

[11] Sawai D, Tamada M, Yokoyama T, Kanamoto T, Hyon S-H, Moon S (2007). Sen’I Gakkaishi.

[12] Sawai D, Tamada M, Kanamoto T (2007). Polym J.

[13] Zhang J, Tashiro K, Tsuji H, Domb A J (2007). Macromolecules.

[14] Kakiage M, Ichikawa T, Yamanobe T, Uehara H, Sawai D (2010). ACS Appl Mater Interfaces.

[15] Uehara H, Karaki Y, Wada S, Yamanobe T (2010). ACS Appl Mater Interfaces.

[16] Yokoyama Y, Uehara H, Yamanobe T (2013). Key Eng Mater.

[17] Uehara H, Nakae M, Kanamoto T, Zachariades A E, Porter R S (1999). Macromolecules.

[18] Nakae M, Uehara H, Kanamoto T, Ohama T, Porter R S (1999). J Polym Sci, Part B: Polym Phys.

[19] Nakae M, Uehara H, Kanamoto T, Zachariades A E, Porter R S (2000). Macromolecules.

[20] Uehara H, Kakiage N, Yamanobe T, Komoto T, Murakami S (2006). Macromol Rapid Commun.

[21] Kakiage M, Yamanobe T, Komoto T, Murakami S, Uehara H (2006). J Polym Sci, Part B: Polym Phys.

[22] Kakiage M, Yamanobe T, Komoto T, Murakami S, Uehara H (2006). Polymer.

[23] Uehara H, Yoshida R, Kakiage M, Yamanobe T, Komoto T (2006). Ind Eng Chem Res.

[24] Kakiage M, Sekiya M, Yamanobe T, Komoto T, Sasaki S, Murakami S, Uehara H (2007). Polymer.

[25] Morioka T, Kakiage M, Yamanobe T, Komoto T, Higuchi Y, Kamiya H, Arai K, Murakami S, Uehara H (2007). Macromolecules.

[26] Kakiage M, Sekiya M, Yamanobe T, Komoto T, Sasaki S, Murakami S, Uehara H (2008). J Phys Chem B.

[27] Kakiage M, Uehara H, Yamanobe T (2008). Macromol Rapid Commun.

[28] He Y, Inoue Y (2003). Biomacromolecules.

[29] He Y, Inoue Y (2004). J Polym Sci, Part B: Polym Phys.

[30] Dong T, He Y, Zhu B, Shin K-M, Inoue Y (2005). Macromolecules.

[31] Dong T, Shin K-m, Zhu B, Inoue Y (2006). Macromolecules.

[32] Shin K-M, Dong T, He Y, Inoue Y (2005). J Polym Sci, Part B: Polym Phys.

[R33] 33Joijode, A. S. Behavior and Properties of Self-Nucleated Poly (ethylene terephthalate) (PET). MS Thesis, North Carolina State University, USA. 2011, p. 41.

[34] Shuai X, Wei M, Porbeni F E, Bullions T A, Tonelli A E (2002). Biomacromolecules.

[35] Ohya Y, Takamido S, Nagahama K, Ouchi T, Ooya T, Katoono R, Yui N (2007). Macromolecules.

[36] Xie D M, Yang K S, Xun W X (2007). Curr Appl Phys.

[37] Mano J F (2008). Macromol Rapid Commun.

[38] Suzuki T, Morikawa J, Hashimoto T, Buividas R, Gervinskas G, Paipulas D, Malinauskas M, Mizeikis V, Juodkazis S (2012). Proc SPIE.

[39] Suzuki T, Takahashi K, Uehara H, Yamanobe T (2013). J Therm Anal Calorim.

[40] Hoidy W H, Ahmad M B, Jaffar Al-Mulla E A, Ibrahim N A B (2010). J Appl Sci.

[41] Aree T, Saenger W, Leibnitz P, Hoier H (1999). Carbohydr Res.

[42] Giordano F, Novak C, Moyano J R (2001). Thermochim Acta.

[43] Ren Z, Dong L, Yang Y (2006). J Appl Polym Sci.

[44] Ljungberg N, Wesslén B (2002). J Appl Polym Sci.

[45] Labrecque L V, Kumar R A, Davé V, MeCarthy S P (1997). J Appl Polym Sci.

[46] Ljungberg N, Andersson T, Wesslén B (2003). J Appl Polym Sci.

[47] Mano J F, Gómez Ribelles J L, Alves N M, Salmerón Sanchez M (2005). Polymer.

[48] Turner J F, Riga A, O’Connor A, Zhang J, Collis J (2004). J Therm Anal Calorim.

[49] Cao X, Mohamed A, Gordon S H, Willett J L, Sessa D J (2003). Thermochim Acta.

[50] Nazhat S N, Kellomäki M, Törmälä P, Tanner K E, Bonfield W (2001). J Biomed Mater Res.

[51] Tan H Y, Widjaja E, Boey F, Loo S C J (2009). J Biomed Mater Res, Part B.

[52] Radjabian M, Kish M H, Mohammadi N (2010). J Appl Polym Sci.

[53] Bertoluzza A, Rossi M, Taddei P, Redenti E, Zanol M, Ventura P (1999). J Mol Struct.

[54] Kister G, Cassanas G, Vert M (1998). Polymer.

[55] Liem H, Cabanillas-Gonzalez J, Etchegoin P, Bradley D D C (2004). J Phys: Condens Matter.

[56] Deerwester S, Dumais S T, Landauer T K, Furnas G W, Harshman R (1990). J Am Soc Inf Sci.

[57] Aach J, Church G M (2001). Bioinformatics.

[58] Yasuniwa M, Iura K, Dan Y (2007). Polymer.

[59] Hart T R, Aggarwal R L, Lax B (1970). Phys Rev B.

